# Age of donor alters the effect of cyclic hydrostatic pressure on production by human macrophages and osteoblasts of sRANKL, OPG and RANK

**DOI:** 10.1186/1471-2474-7-21

**Published:** 2006-03-06

**Authors:** CE Evans, S Mylchreest, JG Andrew

**Affiliations:** 1 Laboratory & Regenerative Medicine, Stopford Building, The Medical School, University of Manchester, Oxford Road, Manchester M13 9PT, UK; 2 Department of Orthopaedic Surgery, Ysbyty Gwynedd, Bangor, LL57 2PW, UK

## Abstract

**Background:**

Cyclic hydrostatic pressure within bone has been proposed both as a stimulus of aseptic implant loosening and associated bone resorption and of bone formation. We showed previously that cyclical hydrostatic pressure influenced macrophage synthesis of several factors linked to osteoclastogenesis. The osteoprotegerin/soluble receptor activator of NF-kappa β ligand /receptor activator of NF-kappa β (OPG/ RANKL/ RANK) triumvirate has been implicated in control of bone resorption under various circumstances. We studied whether cyclical pressure might affect bone turnover via effects on OPG/ sRANKL/ RANK.

**Methods:**

In this study, cultures of human osteoblasts or macrophages (supplemented with osteoclastogenic factors) or co-cultures of macrophages and osteoblasts (from the same donor), were subjected to cyclic hydrostatic pressure. Secretion of OPG and sRANKL was assayed in the culture media and the cells were stained for RANK and osteoclast markers. Data were analysed by nonparametric statistics.

**Results:**

In co-cultures of macrophages and osteoblasts, pressure modulated secretion of sRANKL or OPG in a variable manner. Examination of the OPG:sRANKL ratio in co cultures without pressurisation showed that the ratio was greater in donors <70 years at the time of operation (p < 0.05 Mann Whitney U) than it was in patients >70 years. However, with pressure the difference in the OPG:sRANKL ratios between young and old donors was not significant. It was striking that in some patients the OPG:sRANKL ratio increased with pressure whereas in some it decreased. The tendency was for the ratio to decrease with pressure in patients younger than 70 years, and increase in patients ≥ 70 years (Fishers exact p < 0.01).

Cultures of osteoblasts alone showed a significant increase in both sRANKL and OPG with pressure, and again there was a decrease in the ratio of OPG:RANKL. Secretion of sRANKL by cultures of macrophages alone was not modulated by pressure. Only sRANKL was assayed in this study, but transmembrane RANKL may also be important in this system. Macrophages subjected to pressure (both alone and in co-culture) stained more strongly for RANK on immunohistochemstry than non-pressurized controls and 1,25-dihydroxyvitamin D_3 _(1,25 D_3_) further increased this. Immunocytochemical staining also demonstrated that more cells in pressurized co-cultures exhibited osteoclast markers (tartrate-resistant acid phosphatase, vitronectin receptor and multinuclearity) than did unpressurized controls.

**Conclusion:**

These data show that in co-cultures of osteoblasts and macrophages the ratio of OPG : sRANKL was decreased by pressure in younger patients but increased in older patients. As falls in this ratio promote bone resorption, this finding may be important in explaining the relatively high incidence of osteolysis around orthopaedic implants in young patients. The finding that secretion of OPG and sRANKL by osteoblasts in monoculture was sensitive to hydrostatic pressure, and that hydrostatic pressure stimulated the differentiation of macrophages into cells exhibiting osteoclast markers indicates that both osteoblasts and preosteoclasts are sensitive to cyclic pressure. However, the effects of pressure on cocultures were not simply additive and coculture appears useful to examine the interaction of these cell types.

These findings have implications for future therapies for aseptic loosening and for the development of tests to predict the development of this condition.

## Background

The effect of strain on bone is now well documented [1-3] and it is clear that increased strain generally leads to increased bone formation. A few studies have examined the effect of hydrostatic compression on bone or bone cells [4-10] and similarly found that hydrostatic compression is likely to lead to increased net bone formation. Both cancellous and cortical bone has a structure of multiple interconnected spaces and there is evidence that applied strain leads to changes in intraosseous pressure. It is possible that, biologically speaking, cyclic hydrostatic pressure may be more influential than strain in a dense structural tissue such as bone.

Aseptic loosening and associated bone loss is the commonest complication of orthopaedic implants. Most studies have concentrated on the role of wear particles and the subsequent invasion of the joint space by activated macrophages [11]. Research has shown that elevated hydrostatic pressures develop in aseptic loosening and there is increasing evidence that this may contribute to loosening and macrophage activation [12-18]. The activated macrophages are responsible for the increased bone resorption seen in aseptic loosening, and several studies have demonstrated that they are able to differentiate into osteoclasts [19-22]. There has been debate about whether, in vivo, macrophage rich membranes are responsible for bone resorption per se, or whether this is only performed by osteoclasts which are in turn activated by products of the macrophage. The actual importance of this distinction for possible treatment of aseptic loosening is unclear.

Previous studies in our laboratory demonstrated that cyclical hydrostatic pressure increased macrophage synthesis of several cytokines [23,24], of macrophage-colony stimulating factor (M-CSF), chemokines and prostaglandin E2 [25]. These molecules are known to be important in bone resorption and turnover and in osteoclastogenesis, but the mechanisms by which they exert their effects in vivo (especially in relation to implant loosening) are still poorly understood. In addition, we have shown that cyclical hydrostatic pressure increased macrophage-mediated bone resorption in bone organ cultures [24].

The three molecules, RANK, RANKL and OPG, have now been shown to be pivotal to bone homeostasis [26]. They are intimately involved in osteoclastogenesis [27-30] and studies have shown that alterations to the ratio of OPG:RANKL affect bone turnover [31-33]. We therefore examined the effect of cyclical hydrostatic pressure on secretion of OPG, RANKL and RANK by human macrophages and osteoblasts in vitro. In addition, as these molecules are important in the modulation of osteoclastogenesis, the effect of cyclical hydrostatic pressure on the phenotype of the macrophage was also investigated.

## Methods

### Co-cultures of macrophages and osteoblasts

For co-cultures, 50 mls of blood was taken from donor patients 6 weeks after primary hip replacement. Monocytes were isolated using Lymphoprep density gradient medium (Apogent Discoveries UK) as described previously [23]. An average of 11.3 × 10^6 ^monocytes were isolated per sample. Briefly, peripheral blood mononuclear cells were suspended in Minimum Essential Medium (MEM) and placed in a tissue culture flask (Nunc) for one hour at 37°C with 5% CO_2_. Monocytes were separated from the rest of the cell population by adherence. After removal of non-adherent cells, the adherent cells were scraped off using a cell scraper and resuspended in culture medium (MEM, 20% foetal calf serum (FCS), 1% Penicillin / Streptomycin, 1% Glutamate). Cell numbers were counted and used in the experiments as described below.

Osteoblasts were isolated from human trabecular bone using the explant technique, as described previously [34] using bone samples removed during elective surgery for primary hip replacement. Osteoblasts were seeded onto slices of dentine (100 μm thick, 4.0 mm diameter), at 5 × 10^5 ^cells/ml (1 × 10^5 ^per slice) and placed in 96-well plates (Corning UK) before culturing overnight in Dulbecco's modified Eagles medium (DMEM, Invitrogen UK) supplemented with 20% foetal calf serum (FCS, Invitrogen UK). Human macrophages were then seeded onto the osteoblast layer at a density of 5 × 10^5 ^cells/ml (1 × 10^5 ^cells per slice) and the co-cultures were incubated for a further six days in DMEM supplemented with 20% foetal calf serum, 1% glutamine (Invitrogen UK), 30 ng/ml M-CSF, (R+D Systems UK), 10^-8^M Dexamethasone (Sigma UK) and 10^-7^M 1,25D_3 _(Sigma UK). Three replicate wells per treatment were set up for each experiment and both cell types were from the same donor. Informed consent was obtained from patients before samples were taken for this research, and the study was approved by the Salford LREC.

The co-cultures were subjected to 34.5 × 10^-3^MPa (5psi) cyclical hydrostatic pressure (2 secs on, 2 secs off) for one hour for 4 consecutive days in our unique pressurization apparatus [23], in a 5% CO_2_/balance air environment (B.O.C. Special Gases Ltd UK). Cultures were allowed to rest for 23 hours after pressurization before media were harvested for assay and stored at -20°C before assaying for OPG and sRANKL. Cells on the dentine were stained to determine cell type.

### Culture of macrophages

Monocytes were prepared as above, from normal blood or blood from patients prior to primary hip replacement, prior to use in the following experiment.

Human macrophages were seeded onto slices of dentine at 5 × 10^5 ^cells/ml (1 × 10^5 ^cells per slice) before culturing for a further six days in DMEM supplemented with 20% FCS, 1% glutamine, 30 ng/ml M-CSF, 10^-8^M Dexamethasone and 10^-7^M 1,25D_3_. Three replicate wells per treatment were set up for each experiment.

The cultures were subjected to 34.5 × 10^-3^MPa (5psi) cyclical hydrostatic pressure (2 secs on, 2 secs off) for one hour for 4 consecutive days. Cultures were allowed to rest for 23 hours after pressurization before media were harvested for assay and stored at -20°C before assaying for OPG and sRANKL.

### Culture of osteoblasts

Osteoblasts isolated for the co-cultures were also cultured without macrophages. Cells were seeded onto slices of dentine at a cell density of 5 × 10^5 ^cells/ml (1 × 10^5 ^per slice) and cultured as above for 6 days before subjecting them to pressure (see above). Cultures were allowed to rest for 23 hours after pressurization before media were harvested and stored at -20°C before assaying for OPG and sRANKL.

For all co-culture/macrophage/osteoblast experiments, control cultures (i.e. not subjected to hydrostatic pressure) were included.

### Effect of hydrostatic pressure on OPG and sRANKL secretion

Culture media were assayed directly for sRANKL using a specific ELISA kit (Peprotech EC – human sRANKL ELISA development kit), according to the manufacturers directions. Culture media was assayed for OPG using an in-house ELISA method [35]. A goat anti-human OPG antibody was used (R+D Systems UK) to coat the ELISA plates (Appleton Woods UK). The standard curve consisted of dilutions of recombinant human OPG (Alexis Corp.). A biotinylated goat anti-human OPG antibody (R+D Systems UK) was used with ExtraAvidin peroxidase (Sigma UK) to amplify the signal. OPG binding was visualised using TMB (Pierce Chemical Co.) and optical densities determined at 450 nm. Inter- and intra- assay variation was 12.8% and 7.7% respectively and specificity was confirmed by recovery experiments using heparin-affinity purified human OPG.

For all these experiments, control cultures (i.e. not subjected to hydrostatic pressure) were included.

### Effect of 1,25 dihydroxyvitamin D_3 _on RANK production by macrophages

Monocytes were isolated as described previously [23] from normal blood or blood from patients prior to primary hip replacement. The macrophages thus obtained were cultured as before [23] prior to use in the experiments outlined below. Macrophages were cultured at 5 × 10^5^/ml on Thermanox coverslips (Nunc, UK) in 24-well plates (Corning UK) and incubated in DMEM with 20% FCS and 1% glutamine, for 7 days. 1,25D_3 _was added to the cultures at either 10^-7^M or 10^-9^M one hour before cultures were pressurised at 34.5 × 10^-3^MPa (5.0 psi), (2 secs on, 2 secs off) for one hour [23]. To examine whether the effect of pressure was additive, some cultures were subjected to one cycle of pressure (1 × 1 hour) and others to four cycles (the four cycles being performed on consecutive days). Cultures were allowed to rest for 23 hours after pressurization before macrophages were fixed in 100% ice-cold ethanol (BDH, UK) for two minutes.

Cell cultures were immunostained for RANK with a specific polyclonal antibody to RANK (Santa Cruz Biotechnology, USA; RANK C-20). Briefly, cells were incubated overnight at 4°C with anti-RANK (1/100) and positive staining disclosed using the ABC technique (Vectastain, UK). Control slides were incubated with 1% non-immune serum (Normal horse serum; Vectastain) to check antibody specificity. All slides were stained at the same time to minimise variation in staining intensity.

### Effect of hydrostatic pressure on osteoclast markers

Monocytes were isolated as described previously [25] from the venous blood of healthy volunteers. The macrophages thus obtained were cultured as before [25] prior to use in the experiment outlined below. Macrophages were cultured at 5 × 10^5^/ml on Thermanox coverslips (Nunc, UK) in 24-well plates and incubated in DMEM, 20% FCS, 1% Glutamine, supplemented with 30 ng/ml M-CSF, 10^-8^M Dexamethasone, 30 ng/ml sRANKL and 10^-7^M 1,25D_3_, for 7 days. Cultures were then subjected to hydrostatic pressure at 34.5 × 10^-3^MPa (5.0 psi) (2 secs on, 2 secs off) for one hour.

To examine whether the effect of pressure was additive, cultures were subjected to either one or four cycles of pressure (the four cycles being performed on consecutive days). Cultures were allowed to rest for 23 hours after pressurization before media were harvested and frozen at -20°C. Macrophages were fixed in 100% ice-cold ethanol (BDH, UK) for 2 minutes and stored at -20°C.

Cells were stained for tartrate-resistant acid phosphatase (TRAP) using a commercial kit (Sigma Cat. No.387-A), to demonstrate enzyme activity and multinuclearity. Cells were also immunostained for the vitronectin receptor (VNR) with a specific monoclonal antibody to VNR (Serotech; Cat. No MCA7576). Briefly, cells were incubated overnight at 4°C with anti-VNR (1/100) and positive staining disclosed using the ABC technique (Vectastain, UK). Control slides were incubated with 1% non-immune serum (Normal horse serum; Vectastain) to check antibody specificity.

For all these experiments, control cultures (i.e. not subjected to hydrostatic pressure) of cells from the same population were included.

### Statistical analysis

Statistical analysis was performed on the data, where relevant. To demonstrate the effect of pressure, Friedmanns non-parametric two-way ANOVA followed by the Wilcoxon Signed Ranks Test was used. To demonstrate significance between different age groups, Mann Whitney U and Fishers Exact Test was used.

## Results

### Effect of hydrostatic pressure on OPG and sRANKL secretion

Effect on co-cultures of macrophages and osteoblasts:

Initial analysis of the data, where the results from individual patients were pooled, suggested that pressure did not modulate secretion of sRANKL and OPG by co-cultures (Table 1).

However, calculation of the ratios of OPG:sRANKL for individual patients showed an increase with pressure in some samples and a decrease in others (Table 2). Examination of the effect of donor age showed that the unpressurised OPG:sRANKL ratio was lower in samples from patients over 70 years (Mann Whitney U p < 0.05). (Table 2.) The application of pressure resulted in this difference becoming much less evident (p = 0.94). The difference between OPG:sRANKL (with pressure) and (without pressure) was smaller or negative in patients <70 years (Mann Whitney U p < 0.05) (Table 3). Pressure was more likely to cause an increase in the ratio in patients of 65 or over, and a decrease in younger patients (p < 0.01 Fishers exact test).

Effects on monocultures of osteoblasts:

For osteoblasts (from the same cell population) cultured without macrophages, pressure modulated secretion of both sRANKL and OPG. Pressure increased secretion of sRANKL by 73% and this increase was statistically significant (p = 0.002) (Wilcoxon Signed Ranks test); OPG secretion was increased by 89% and this increase was statistically significant (p = 0.034) (Wilcoxon Signed Ranks test) (Table 1). No change in the ratio of OPG:sRANKL was detected with pressure (65.2 ± 69.2 [no pressure] and 43.7 ± 42.6 [with pressure]).

Effects on monocultures of macrophages:

Pressure had no effect on secretion of sRANKL by macrophages cultured without osteoblasts (Table 1) (p = 0.69) (Wilcoxon Signed Ranks test) and OPG was not detectable in most of these cultures.

It was noticeable that basal secretion of sRANKL in the monocultures was substantially reduced and that basal secretion of OPG increased, compared with the co-cultures (Table 1). The application of pressure to the cultures increased the total secretion of sRANKL in the osteoblast monocultures to that seen in the co-cultures, but this was not seen in the macrophage monocultures. It is notable that pressure increased total OPG secretion by monocultures of osteoblasts to above that seen in the pressurized co-cultures (Table 1).

### Effect of hydrostatic pressure on RANK in macrophage cultures

**Figure 1:**
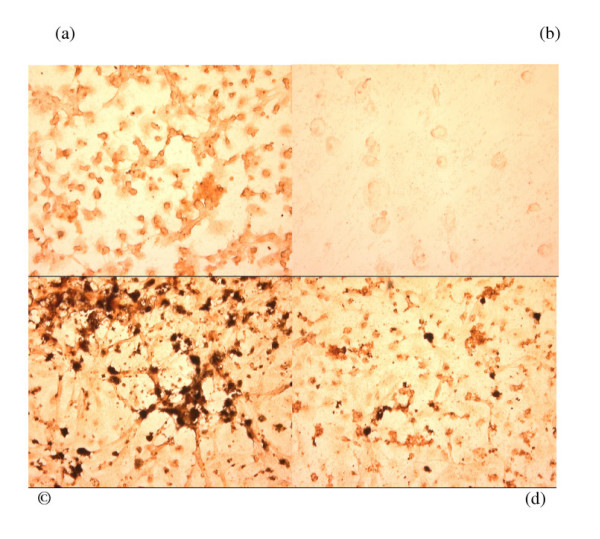
**Effect of hydrostatic pressure and 1,25D_3 _on synthesis of RANK by cultured human macrophages**. Macrophages were cultured at 5 × 10^5^/well for 7 days. 1,25D_3 _(10^-7^M or 10^-9^M) was added and cultures were subjected to one cycle of pressure (34.5 × 10^-3^MPa). Macrophages were immunostained for RANK with a specific polyclonal antibody. Cells exposed to pressure and 10^-7^M 1,25D_3 _(a) synthesised more RANK than cells exposed to pressure and 10^-9^M 1,25D_3 _(b). Cultures supplemented with 1,25D_3 _synthesised more RANK than control cultures without 1,25D_3 _(c). MP in pressurised synthesised more RANK than in control cultures without pressure (d).

**Figure 2:**
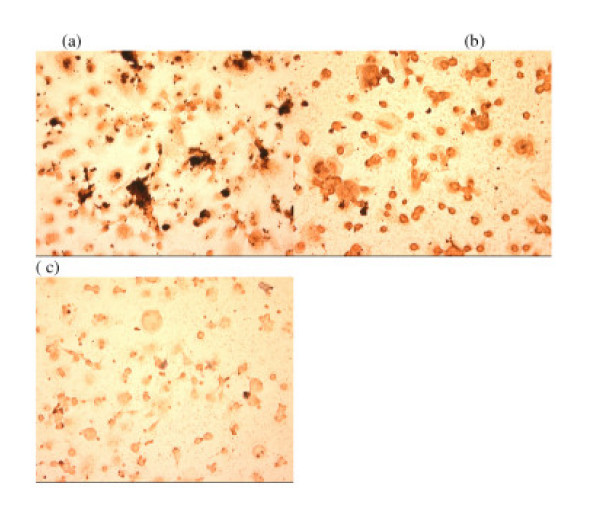
**Effect of 1,25D_3 _alone on synthesis of RANK by cultured human macrophages**. Macrophages were cultured at 5 × 10^5^/well for 7 days. 1,25D_3 _(10^-7^M or 10^-9^M) was added. Macrophages were immunostained for RANK with a specific polyclonal antibody. Cells exposed to 10^-7^M 1,25D_3 _(a) synthesised more RANK than cells exposed to 10^-9^M 1,25D_3 _(b). Cultures supplemented with 1,25D_3 _synthesised more RANK than control cultures without 1,25D_3 _(c).

Macrophages cultured without pressure (controls) synthesised RANK (Fig 1) and, subjectively speaking, cultures exposed to pressure (Fig 1a.) appeared to be stained more intensely for RANK than controls (Fig 1b). This synthesis was further modulated when macrophages were incubated with 1,25D_3, _as RANK staining was more intense in cultures incubated with 10^-7^M (Fig 1c.) compared to the lower concentration, 10^-9^M (Fig 1d.). Even without pressure, staining was more intense in macrophages cultured with 1,25D_3 _(Fig 2a,b) than those cultured without (Fig 2c.) and again staining was more intense in with 10^-7^M (Fig 2a.) compared to 10^-9^M (Fig 2b.).

**Figure 3:**
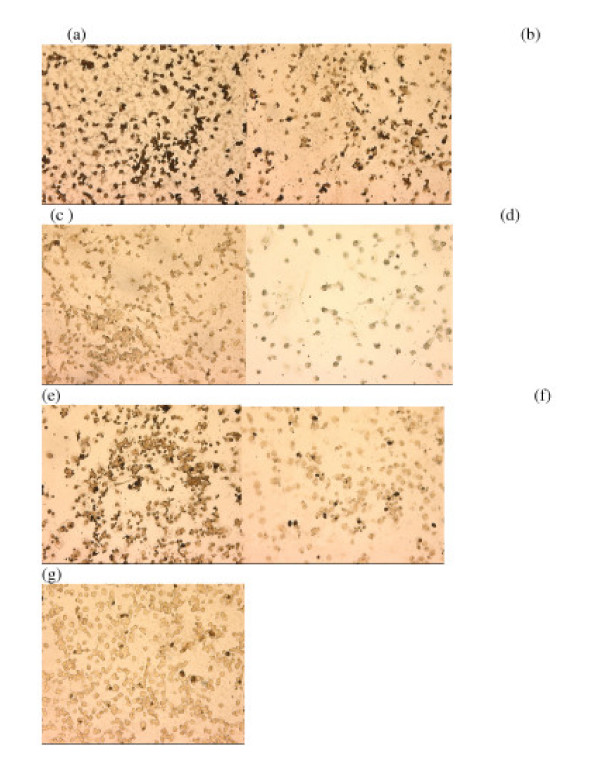
**Cumulative effect of pressure on synthesis of RANK by cultured human macrophages**. Macrophages were cultured at 5 × 10^5^/well for 7 days. 1,25D_3 _(10^-7^M or 10^-9^M) was added and cultures were subjected to four cycles of pressure (34.5 × 10^-3^MPa). Macrophages were immunostained for RANK with a specific polyclonal antibody. Cells exposed to pressure and 10^-7^M 1,25D_3 _(a) synthesised more RANK than cells exposed to pressure and 10^-9^M 1,25D_3 _(b). Cultures supplemented with 1,25D_3 _synthesised more RANK than control cultures without 1,25D_3 _(c). Pressurised MP synthesised more RANK than control cultures without pressure (d). In unpressurized cultures, MP synthesis of RANK again varied with the stimulus; 10^-7^M 1,25D_3 _(e); 10^-9^M 1,25D_3 _(f); no 1,25D_3 _(g).

Cyclical hydrostatic pressure appeared to have a cumulative effect on RANK synthesis, with staining appearing more intense in cultures exposed to four cycles of pressure (Fig 3.), compared to those only exposed to one cycle (Fig 1.). In addition, RANK staining was more intense in pressurized cultures (Fig 3a–c) compared to static controls (Fig 3d–g), regardless of the addition of 1,25D_3_. Again, RANK staining was more intense in macrophages incubated with 1,25D_3 _(Fig 3a,b) than control cultures (Fig 3c) and staining intensity was greater in cultures exposed to the higher level of 1,25D_3, _10^-7^M, compared to 10^-9^M (3a,b.).

The intensity of the RANK immunostaining was semi-quantified by grading from a score of no positively stained cells (-), some positively stained cells (+/-), to all cells positively and densely stained (+++) (Table 3.).

### Effect of hydrostatic pressure on OC markers

**Figure 4:**
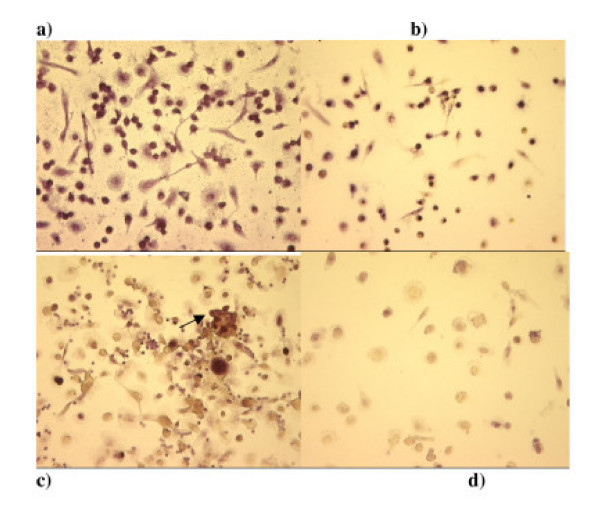
**Effect of hydrostatic pressure on the number of TRAP positive and multinucleated cells**. MP (5 × 10^5^/ml) from healthy volunteers were cultured at 5 × 10^5 ^cells/ml on Thermanox coverslips in supplemented medium for 7 days before pressurisation (34.5 × 10^-3^MPa) (5.0 psi) for four cycles of pressure. MP were fixed and stained for TRAP; a) with pressure; b) without pressure. More multinucleated cells (arrows) were seen with pressure c) than without d).

The number of TRAP positive cells was increased in cultures exposed to cyclical hydrostatic pressure (Figure 4a) compared to static controls (b). In addition, although only a small proportion of the cells were multinucleated, there were more multinucleated cells in pressurized cultures (Fig 4c) compared to controls (Fig 4d).

**Figure 5:**
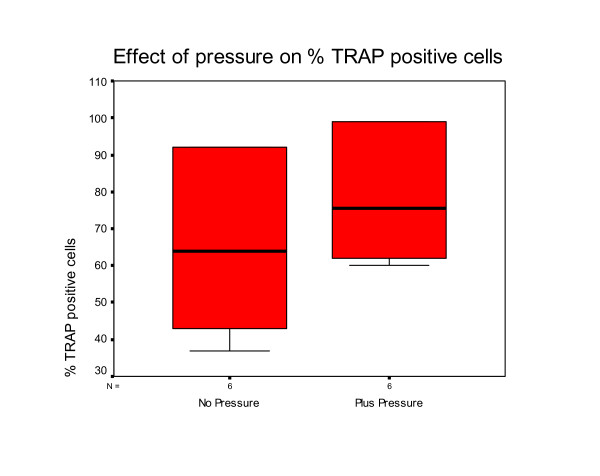
**Effect of hydrostatic pressure on the percentage of TRAP positive cells**. MP (5 × 10^5^/ml) from healthy volunteers were cultured at 5 × 10^5 ^cells/ml on Thermanox coverslips in supplemented medium for 7 days before pressurisation (34.5 × 10^-3^MPa) (5.0 psi) for four cycles of pressure. MP were fixed and stained for TRAP. For each sample, the number of positive and negative cells in 5 random microscope fields was counted, for each of three replicates. Results are the mean and SD of 5 separate experiments.

The total number of TRAP positive and TRAP negative cells per culture was counted using a light microscope (Vickers) and a × 20 objective. Exposure to cyclical pressure produced a larger number of TRAP positive cells compared to the static control cultures. The mean number of TRAP positive cells in pressurized cultures was 173 ± 86 (SD) compared to only 128 ± 88 (SD) in non-pressurized cultures. These results are calculated from cell counts from 5 separate experiments, with three replicate wells per experiment (n = 15). Statistical analysis demonstrated that the increase was highly statistically significant (Wilcoxon Signed Ranks Test; p < 0.0001). The percentage of TRAP positive cells in pressurized cells compared to static controls (Fig 5) was statistically significantly different (Wilcoxon Signed Ranks Test; p = 0.026). The percentage increase in the number of TRAP positive cells induced by pressure was variable however, with increases within the range 9–88%, with a mean increase of 28 ± 12 (SD).

**Figure 6:**
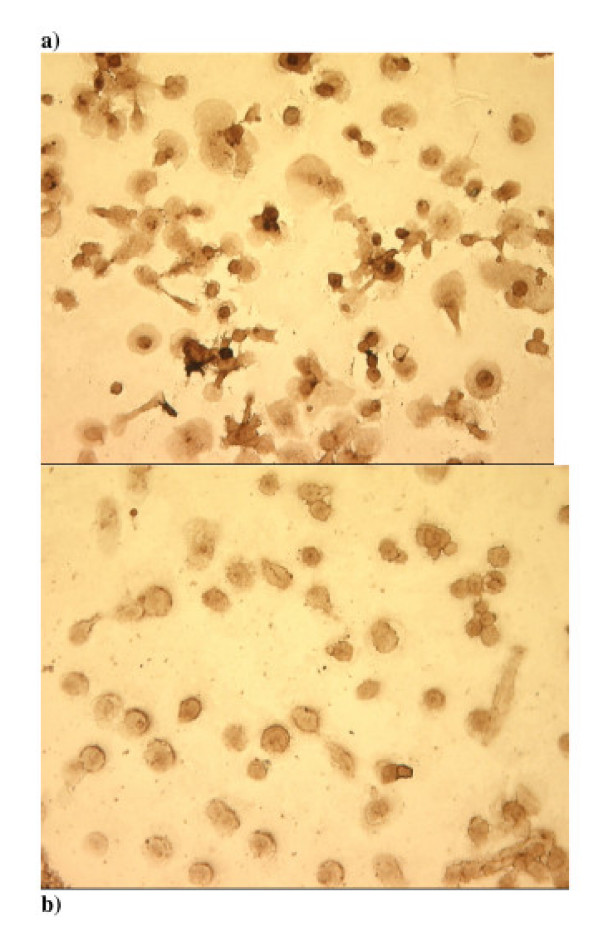
**Effect of hydrostatic pressure on the number of VNR positive cells**. MP (5 × 10^5^/ml) from healthy volunteers were cultured at 5 × 10^5 ^cells/ml on Thermanox coverslips in supplemented medium for 7 days before pressurisation (34.5 × 10^-3^MPa) (5.0 psi) for four cycles of pressure. MP were fixed and immunostained for the VNR; a) with pressure; b) without pressure.

Immunostaining cultures for the VNR demonstrated that the number of VNR positive cells was also increased by pressure (Fig 6a) compared to controls (6b).

## Discussion

Three molecules, OPG, RANKL and RANK, play a vital role in controlling bone homeostasis [26]. Osteoclastogenesis is initiated and maintained by at least two key factors produced locally in bone, M-CSF and RANKL. RANKL is found both as a transmembrane molecule on osteoblasts, which also secrete OPG, and as a secreted molecule, sRANKL. OPG opposes RANKL and blocks osteoclastogenesis. RANK is found on osteoclast-like cells and mononuclear cells in bone [33] and other tissues [36,37]. The balance between OPG, RANKL and RANK is crucial to bone homeostasis [38] as the ratio between OPG and RANKL modulates osteoclast activity and has been shown to vary during bone turnover [31-33,39]. Increases in OPG:RANKL would be expected to favour bone formation, and decreases to favour resorption.

To date, only a handful of studies have addressed the role of RANKL in aseptic loosening [40-42], whilst the importance of OPG has only recently been examined in this context [43]. The response to mechanical load *vis a vis *RANKL and OPG is variable. Strain has been shown to increase expression of RANKL mRNA in rat periodontal ligaments in vivo [44]. In vitro, strain had no effect on synthesis of RANKL protein by periodontal ligament cells [45] although OPG expression was increased [45]. Bone stromal cells respond differently to strain, which decreased RANKL mRNA in one study [46] and increased it in another [47]. To the authors' knowledge, no studies to date have examined the effect of hydrostatic pressure on OPG or sRANKL in macrophages or osteoblasts.

The results reported here demonstrate that in cocultures of osteoblasts and macrophages from the same donor patient, the OPG:sRANKL ratio is affected both by age and by cyclic pressure. Without pressure, young patients (<70 years at operation) had higher OPG:sRANKL ratios. The effect of pressure on this was different in young and old patients, with young patients tending to show a fall in OPG:sRANKL and older patients showing a rise. After application of pressure there was no longer a significant difference between patients above and below 70 years.

Other groups have found a correlation between age and changes in the ratio of RANKL:OPG [31]. Most patient studies have demonstrated an increase in circulating OPG with age [48-51] together with a decrease in sRANKL [48,51]. Conversely, an in vitro study using murine bone marrow cells co-cultured with pre-osteoclasts demonstrated that, although osteoclastogenesis increased with age, the levels of RANKL mRNA increased whilst mRNA for OPG decreased [52]. It is of interest that several pathologies have been linked to alterations in OPG and RANKL. The ratio of RANKL:OPG was increased in bone from elderly fracture patients [53,54] and low levels of RANKL have been suggested as a predictor of fracture [55]. In addition, in patients with rheumatoid arthritis, RANKL polymorphisms were associated with an earlier age of disease onset [56].

The finding that the effect of pressure on OPG:sRANKL in cocultures was different in young and old patients was unexpected. Young patients, who would normally be expected to have a bone forming response to activity (and hence to changes in intraosseous pressure), proved to often have a fall of OPG:sRANKL with pressure. This may be because our co-culture setup has a high ratio of macrophages (osteoclast precursors) to osteoblasts. The differing secretion of the mediators in monoculture and co-culture strongly suggest that the relative numbers of macrophages and osteoblasts will influence the OPG:sRANKL ratio. The setup of the co-cultures was designed to model the bone/membrane interface of a loosening membrane, and it is likely that it does not correspond to a normal bone surface. The proportions of macrophages and osteoblasts are more similar to the loosening membrane situation than to the surface of a normal piece of bone. The change in the effect of cyclic pressure with age may help explain why results of hip replacements are consistently better in older people (Swedish Hip Register 2003). This has previously been attributed to reduction in levels of activity (and hence implant wear) with age, but our findings indicate that young patients may be biologically more susceptible to the development of osteolysis and bone resorption in this situation. This finding may have value in developing tests predictive of implant loosening in young patients.

In osteoblasts cultured alone, secretion of both sRANKL and OPG was significantly increased under pressure and the mean ratio of OPG:sRANKL was decreased 49% in osteoblast monoculture. Pressure had no effect on secretion of sRANKL by MP monoculture and these cells secreted no detectable OPG. There is a theoretical possibility that, in these cultures, complexes may form between OPG and sRANKL. Whilst we know that TRAIL and RANKL inhibit the OPG assay (personal communication) no data is available on binding to such complexes for the sRANKL assay kit.

Comparison between co cultures and monocultures of detectable product (sRANKL or OPG) and of the mean ratio of OPG:sRANKL suggests a feedback mechanism could be operating between these two cell types in vitro and that pressure modulates this cell-cell communication. Active osteoblastic activity has previously been reported at the bone surface adjacent to loosening membranes [57], and, as our data demonstrated that pressure affected the ratio between OPG and sRANKL it is tempting to speculate that a similar effect may be produced in the in vivo situation. This suggested scenario does not take into account the contribution of transmembrane RANKL, which, for technical reasons, was not assayed in this study.

In addition, immunostaining of MP for RANK showed a clear increase in RANK after pressurization, compared to controls. In addition, the effect of pressure on RANK synthesis was cumulative, as RANK synthesis was increased when the number of loading cycles was increased. It is of interest that RANK synthesis was also increased when MP were cultured with 1,25 D_3 _(a factor strongly implicated in bone resorption) [58], as we have shown that pressure increases synthesis of 1,25 D_3 _by MP [59]. Other studies have also shown that 1,25 D_3 _increased RANK expression in MP [60]. In addition, expression of RANK has been shown to be elevated in peri-implant tissue in aseptic loosening, aiding osteoclast differentiation [40] and, as RANK demonstrates a autoregulatory positive feedback system, this could further enhance osteoclast differentiation in this tissue [60].

Cyclical hydrostatic pressure also modulated other markers on monocultures of human MP, moving them towards an osteoclast-like phenotype. After exposure to five sets of cyclical pressure, the majority of cells were positive for both TRAP and VNR. To our knowledge, this is the first time that cyclical hydrostatic pressure has been shown to influence MP differentiation in isolated MP. Others [61] have demonstrated that static compression inhibited osteoclast formation, but these studies were done using mouse marrow, where a mixed cell population was present and therefore no direct comparison can be made.

Other stimuli linked to osteoclastogenesis (1,25 D_3_, RANKL or osteoblasts themselves) increased the number of positive cells for these markers, when compared to controls. Exposure to the additional stimulus of hydrostatic pressure further increased the numbers of positive cells. However, very few multinucleated cells were visible, indicating that cell fusion, the end-stage of osteoclast differentiation, was not stimulated by pressure over the time scale of these studies.

## Conclusion

An age-related difference in the ratio of OPG:sRANKL was found in co-cultures of osteoblasts and macrophages and cyclic hydrostatic pressure modulated this ratio, causing a reduction in younger patients. In monocultures, pressure increased OPG:sRANKL secretion by osteoblasts and RANK synthesis by macrophages. The macrophage phenotype was also modulated, generating osteoclast-like cells. In summary, pressure modulates cell activity, disturbing homeostasis and favouring osteolysis; the balance of the system differs between young and old patients.

## Competing interests

The author(s) declare that they have no competing interests.

## Authors' contributions

CEE conceived and designed the study in conjunction with JGA. JGA provided the clinical samples. CEE ran the study overall, interpreted the data, performed statistical analyses and drafted the manuscript. JGA also performed statistical analyses and helped with interpretation of data and helped with drafting the manuscript. SM carried out all the tissue culture, performed all the assays, analysed the data, coordinated the study and contributed to the manuscript.

## Pre-publication history

The pre-publication history for this paper can be accessed here:



## Figures and Tables

**Table 1:** The effect of pressure on secretion of sRANKL and OPG by macrophages and osteoblasts cultured alone or in co-culture

	**Co-cultures of MP & OB**	**OB**	**MP**
**sRANKL (pg/ml)**			
**+ P**	672 ± 180	703 ± 118	264 ± 39
**- P**	878 ± 193	421 ± 61	270 ± 45
**OPG (ng/ml)**			
**+ P**	9.7 ± 2.7	23.6 ± 4.2	0
**- P**	8.1 ± 1.75	12.5 ± 4.3	0

**Table 2:** The effect of pressure on ratio of OPG : sRANKLis influenced by the age of the donor

**Patient**	**Age**	**OPG: sRANKL - P**	**OPG: sRANKL + P**	**+ P : - P**
MK	40.97	8.38	0	0
AH	53.76	20.6	19.4	0.941748
MM	57.06	6.2	11	1.774194
SS	59.41	150	25	0.166667
CW	64.33	42.2	17.8	0.421801
SR	65.5	53.7	59.2	1.102321
DH	65.72	15.4	19.5	1.266234
SC	72.44	6.59	9.46	1.435508
KN	72.76	11.28	22.1	1.95922
JR	75.38	3	18	6
MI	77.58	12.9	30	2.325581
LM	81.5	5.6	20	3.571429
GB	81.61	3.24	3.92	1.209877

**Table 3:** The effect of age on direction of change of OPG:sRANKL with pressure.  cases decreased the OPG:sRANKL ratio with cyclic pressure, while  cases increased the ratio

	<65 (mean 55 y)	≥65 (mean 74 y)	P < 0.01 Fishers exact test
	4	0	
	1	8	

Osteoblasts were seeded onto slices of dentine at 5 × 10^5 ^cells/ml, placed in 96-well plates and cultured overnight. Macrophages were seeded onto the osteoblast layer at 5 × 10^5 ^cells/ml and cultured for a further six days in DMEM supplemented with M-CSF (30 ng/ml), Dexamethasone (10^-8^M) and 1,25D_3 _(10^-7^M). Three replicate wells per treatment were set up for each experiment and both cell types were from the same donor. The effect of pressure ("P") and age on secretion of sRANKL and OPG was compared using the Mann Whitney U test.

**Table 4:** Synthesis of RANK by human macrophages is modulated by both hydrostatic pressure and 1,25-dihydroxy vitamin D_3_

**No. Cycles/ Experiment period (days)**	**No Pressure**			**Plus Pressure**		
	**- D_3_**	**+ D_3 _(10-9*M*)**	**+ D_3 _(10-7*M*)**	**- D_3_**	**+ D_3 _(10-9*M*)**	**+ D_3 _(10-7*M*)**
**1**	-/+	+	+/++	+	+/++	++
**4**	+	+/++	++	+	++	+++

MP were cultured as described in text and and cultures were scored for the proportion of RANK-positive cells, where "+/-" is some positive cells, "+" all positive cells, "+/++"all positive cells & staining more intense and "+++" is the most intense staining. Results are the means from separate experiments.
